# Oil-Painting Style Classification Using ResNet with Conditional Information Bottleneck Regularization

**DOI:** 10.3390/e27070677

**Published:** 2025-06-25

**Authors:** Yaling Dang, Fei Duan, Jia Chen

**Affiliations:** 1 School of Art and Design, Shanxi University of Electronic Science and Technology, Linfen 041000, China; chenjia@sxdzkj.edu.cn; 2 Department of Fine Arts and Craft Design, Yuncheng University, Yuncheng 044030, China

**Keywords:** oil-painting style classification, conditional information bottleneck, matrix-based Rényi’s α-order entropy functional

## Abstract

Automatic classification of oil-painting styles holds significant promise for art history, digital archiving, and forensic investigation by offering objective, scalable analysis of visual artistic attributes. In this paper, we introduce a deep conditional information bottleneck (CIB) framework, built atop ResNet-50, for fine-grained style classification of oil paintings. Unlike traditional information bottleneck (IB) approaches that minimize the mutual information I(X;Z) between input *X* and latent representation *Z*, our CIB minimizes the conditional mutual information I(X;Z∣Y), where *Y* denotes the painting’s style label. We implement this conditional term using a matrix-based Rényi’s entropy estimator, thereby avoiding costly variational approximations and ensuring computational efficiency. We evaluate our method on two public benchmarks: the Pandora dataset (7740 images across 12 artistic movements) and the OilPainting dataset (19,787 images across 17 styles). Our method outperforms the prevalent ResNet with a relative performance gain of 13.1% on Pandora and 11.9% on OilPainting. Beyond quantitative gains, our approach yields more disentangled latent representations that cluster semantically similar styles, facilitating interpretability.

## 1. Introduction

Oil painting, as a medium, has served as a cornerstone of artistic innovation for centuries, capturing the interplay of technique and cultural zeitgeist across movements, from the idealized harmonies of the Renaissance to the visceral impasto of the Baroque. Yet, the taxonomic boundaries of these styles often reside in granular, curatorially honed details: the directional thrust of a brushstroke, the optical blending of layered glazes, or the spatial rhythm of compositional geometry [1,2]. While human experts leverage decades of visual literacy to decode these cues, the rise of digitized art collections, now spanning millions of high-resolution works—demands scalable, objective tools to map stylistic lineages, detect forgeries [3,4], and democratize access to art historical narratives [5,6].

Convolutional neural networks (CNNs), notably ResNet [7], have revolutionized image recognition by hierarchically encoding texture and shape. However, the direct application of ResNet to fine-grained style classification faces a critical mismatch: unlike natural objects, artistic styles are defined not by semantic content but by stylometric signatures, such as subtle variations in brushwork, localized edge contrasts, and chromatic temperature. These discriminative cues often lie in mid-to-high spatial frequency bands, making them particularly vulnerable to suppression by conventional pooling operations or invariant feature learning strategies [1]. While the information bottleneck (IB) principle [8] provides a theoretically grounded framework for learning minimal sufficient representations, *unconditional* variants [9] compress input features without regard to class-specific structure, often discarding style-relevant information along with redundant background content. This issue is particularly problematic in art domains, where inter-class ambiguity (e.g., between Impressionism and Romanticism) and intra-class diversity demand careful preservation of class-conditional details. To address this, we adopt the *conditional information bottleneck* (CIB) formulation, which explicitly encourages representations that retain only the information relevant for distinguishing between style labels, i.e., maximizing I(Z;X∣Y). In doing so, CIB enables the model to filter out nuisance factors unrelated to the target style label (such as background, canvas color, and object semantics), retain only the information that helps distinguish among styles (for example, brushstroke directionality in Expressionism, pointillist dot patterns in Post-Impressionism, or chiaroscuro lighting contrasts in Baroque), and mitigate overfitting to incidental patterns that may vary across artists but are irrelevant to style categories. Furthermore, compared to variational alternatives [9], CIB facilitates a more faithful disentanglement of overlapping styles by conditioning the compression process on the style label, thereby aligning the learned latent space with the stylistic structure of the dataset.

We address these limitations with a conditional information bottleneck (CIB) framework, implemented on a ResNet-50 backbone [7]. By inserting a bottleneck layer after the last block of the Conv5_X stage—a strategic locus preserving brushstroke-level texture before high-level semantic abstraction—we optimize a novel loss that minimizes the mutual information I(X;Z|Y) between input images *X* and latent codes *Z*, *conditioned* on style labels *Y*. This forces the model to discard *only* features statistically independent of style, unlike unconditional IB’s indiscriminate compression. Crucially, we sidestep variational approximations by computing I(X;Z|Y) directly via a matrix-based Rényi’s entropy estimator [10], which quantifies entropy from the eigenspectrum of Gram matrices derived from latent embeddings. This non-parametric approach not only avoids variational biases but also enables end-to-end training without auxiliary networks, yielding both efficiency and interpretability.

Validation on two large-scale benchmarks underscores our gains: the Pandora dataset (7740 images across 12 movements, including Hellenistic frescoes and Romantic landscapes) [11] and the OilPainting corpus (19,787 works spanning 17 styles from Early Netherlandish to Post-Impressionism) [12]. Our CIB-ResNet50 achieves 64.8% accuracy on Pandora, a 7.5% absolute gain over the vanilla ResNet-50 (57.3%), and outperforms both variational IB (61.2%) [9] and deterministic IB (61.6%) [13]. Qualitatively, t-SNE visualizations reveal that CIB induces label-coherent clusters even for stylistically proximate movements (e.g., Baroque vs. Rococo), whereas baseline embeddings exhibit overlapping distributions.

Our key contributions are as follows:

- Label-Conditioned Feature Compression. A CIB mechanism that selectively prunes style-irrelevant features while preserving discriminative brushwork, color, and compositional cues, formalized through conditional mutual information.
- Non-Parametric Entropy Estimation. A training pipeline using Rényi’s α-order entropy functional to compute I(X;Z|Y) without distributional assumptions and variational bounds, improving fidelity and eliminating auxiliary networks.
- Empirical Superiority. State-of-the-art accuracy and adversarial robustness on two oil-painting benchmarks, with ablation studies confirming CIB’s robustness to style granularity.

## 2. Background Knowledge and Related Work

### 2.1. Machine Learning Approaches for Oil-Painting Style Classification

Style classification has long been a focus of both traditional image-processing and deep learning research. Early approaches to painting-style analysis relied on handcrafted features designed to capture brushwork, color palettes, and texture. For instance, Gao et al. [14] applied sparse coding to grayscale patches in order to model local structural patterns for style discrimination. Liu et al. [15] showed that simple statistics of color histograms, such as palette entropy, can effectively distinguish painters. Berezhnoy et al. [16] employed Gabor-basis energy combined with normalized mutual information to quantify stylistic variations in Van Gogh’s oeuvre. More recently, Qian et al. [17] introduced a multi-entropy framework that jointly models block, color, and contour entropy to capture complementary cues of color, composition, and shape. Although these methods achieve competitive results, their dependence on grayscale inputs, manually selected transforms, or hand-tuned entropy measures limits their capacity to capture subtle, higher-order style characteristics inherent in complex oil paintings.

With the advent of convolutional neural networks (CNNs), researchers shifted toward end-to-end style learning. Bai et al. [18] introduced a custom CNN to extract deep style features and evaluate inter-style similarity via an information-bottleneck distance. Early attempts on the Painting-91 dataset [19] applied standard image-classification CNNs [20] to artwork classification, while Folego et al. [21] demonstrated that selecting the patch with the highest confidence score outperforms traditional voting schemes. Nanni et al. [22] showed that combining features from multiple CNN layers yields better style, artist, and architectural classification than using only the top layer, and Peng et al. [23] leveraged multiple CNNs to capture multi-scale representations. Kim et al. [24] further improved accuracy by incorporating visualized depth information of brushstrokes. Menis et al. [25] employ ensemble learning to improve classification performance. More recently, Zhang et al. [26] and Wang et al. [27] confirmed that ResNet-50 [7] provides a strong baseline for oil-painting style classification. Although deep models eliminate the need for handcrafted filters, they often conflate style-relevant cues with semantic content, such as mistaking impasto textures for foliage, and do not explicitly regulate the amount of style-specific information retained. We argue that the key to improving the generalization performance of oil-painting style classification is not to incorporate multi-scale features [23] or to combine predictions from multiple networks [25], but rather to intelligently suppress redundant information in the extracted features. Specifically, representations that include background elements, canvas color, or object semantics may mislead the model, as these factors are often irrelevant to the stylistic identity of the painting and should therefore be compressed. Two challenges, therefore, remain: (1) how to learn features that capture genuine stylistic differences across diverse artistic movements, and (2) how to control and quantify the compression of style-irrelevant information in learned representations.

The information bottleneck (IB) framework formalizes representation learning as an explicit trade-off between compressing the input *X* and preserving information about the target *Y* [8]. Recent extensions of IB parameterize this objective with deep neural networks, including the variational information bottleneck (VIB) [9] and the nonlinear information bottleneck (NIB) [28]. Empirically, IB methods have been shown to improve generalization in domains such as image classification [29], signal classification [30], text classification [31], and robotics [32], supported by strong theoretical guarantees [33]. However, IB approaches remain largely unexplored in the analysis of oil paintings, where variational approximations can become unstable when disentangling overlapping style attributes (e.g., Baroque versus Rococo brushwork).

Our conditional information bottleneck (CIB) approach addresses these challenges by minimizing the conditional mutual information I(X;Z∣Y) using a matrix-based Rényi’s entropy estimator, rather than a variational bound. This explicit regularization forces the latent representation *Z* to retain only style-predictive information, thereby bridging classical entropy-driven style metrics [15,17] and modern deep learning. As a result, CIB delivers robust, interpretable feature compression tailored to the unique texture and compositional dynamics of oil paintings.

### 2.2. Information Bottleneck Principle in Deep Neural Networks

Suppose we have two random variables, *X* and *Y*, linked through their joint probability distribution p(X,Y). We introduce a latent variable *Z*, which serves as a compressed summary of *X*, while preserving the dependency structure Z↔X↔Y. The goal of the information bottleneck (IB) principle is to learn a probabilistic encoder q(z|x) that captures as much relevant information about *Y* as possible, measured by the mutual information I(Z;Y), while discarding irrelevant details from *X* by limiting I(X;Z). Formally, the problem can be expressed as:

(1) $$\max_{q(z|x)}\;I\left(Z;Y\right)\;\mathrm{subject}\;\mathrm{to}\;I\left(X;Z\right)\leq \delta ,$$

where δ controls how much information from the input is retained in the compressed representation.

Rather than handling this constraint explicitly, the IB objective is often reformulated into a single trade-off function:

(2) $$\min_{q(z|x)}\;L_{\mathrm{IB}}=-I\left(Z;Y\right)+\beta I\left(X;Z\right),$$

where the parameter β adjusts the trade-off between compression and predictive performance. A higher β encourages stronger compression, potentially at the cost of reduced predictive power.

The IB principle has both practical and theoretical impacts to DNNs. Practically, it can be formulated as a learning objective (or loss function) for deep models. When parameterizing IB with a deep neural network, *X* denotes the input variable, *Y* denotes the desired output (e.g., class labels), *Z* refers to the latent representation of hidden layers.

However, optimizing the IB Lagrangian is usually difficult, as it involves calculating mutual information terms. Recently, several works [9,28,34,35] have been proposed to derive some lower or upper bounds to approximate the true mutual information values. The prediction term I(Z;Y) is always approximated with the cross-entropy loss. The approximation to I(X;Z) differs for each method. For variational IB (VIB) [9] and similar works [36], I(X;Z) is upper bounded by:

(3) $$\begin{array}{cc} I(X;Z) & =E_{p(x,z)}\log p\left(z|x\right)-E_{p(z)}\log p\left(z\right)\\ \; & \leq E_{p(x,z)}\log p\left(z|x\right)-E_{p(z)}\log v\left(z\right)\\ & =D_{\mathrm{KL}}\left(p\left(z|x\right);v\left(z\right)\right), \end{array}$$

where *v* is some prior distribution such as Gaussian. Depending on application contexts, I(X;Z) can also be measured by the mutual information neural estimator (MINE) [37], which requires training an extra network network to optimize a lower bound of mutual information. More recently, [38] suggests estimating I(X;Z) in a non-parametric way by utilizing the Cauchy–Schwarz divergence quadratic mutual information [39].

Theoretically, it was argued that, even though the IB objective is not explicitly optimized, deep neural networks trained with cross-entropy loss and stochastic gradient descent (SGD) inherently solve the IB compression–prediction trade-off [40,41]. The authors also posed the information plane (IP), i.e., the trajectory in $R^{2}$ of the mutual information pair {I(X;Z),I(Y;Z)} across training epochs, as a lens to analyze dynamics of learning of deep neural networks. According to [40], there are two training phases in the common SGD optimization: an early “fitting” phase, in which both I(X;Z) and I(Z;Y) increase rapidly, and a later “compression” phase, in which there is a reversal such that I(X;Z) continually decrease. This work attracted significant attention, culminating in many follow-up works that tested the proclaimed narrative and its accompanying empirical observations. To date, the “fitting-and-compression” phenomena of the layered representation *Z* have been observed in other types of deep neural networks, including the multilayer perceptrons (e.g., [40,42]), the autoencoders (e.g., [43]), and the CNNs (e.g., [44]). More recently, Kawaguchi et al. [33], Dong et al. [45] formally established the first generalization error bound for the IB objective, showing that an explicit compression term, expressed as either I(X;Z) or I(X;Z∣Y), can improve generalization. However, their conclusions are drawn solely from evaluations on standard image classification benchmarks such as MNIST and CIFAR-10. In contrast, our results, obtained in a completely new application domain and on two significantly more challenging oil painting datasets, offer complementary empirical evidence supporting the claim that the IB principle can improve generalization.

## 3. Methodology

### 3.1. Deep Conditional Information Bottleneck for Oil-Painting Style Classification

The original IB objective improves generalization by minimizing the mutual information I(X;Z). In this work, we adopt an alternative information bottleneck objective, known as the conditional information bottleneck (CIB) [46], formulated as follows:

(4) $$\begin{array}{c} \min_{q(z|x)}L_{IB}=-I\left(Z;Y\right)+\beta I\left(X;Z|Y\right), \end{array}$$

in which the compression term I(X;Z) is replaced by the conditional mutual information (CMI) I(X;Z|Y).

Adopting the conditional mutual information I(X;Z∣Y) in place of the unconditional term I(X;Z) offers several key advantages. Unlike I(X;Z), which can only be minimized by destroying all input information, I(X;Z∣Y) admits a zero minimum—precisely satisfying the minimum necessary information (MNI) criterion by discarding only style-irrelevant variations while fully preserving style-predictive features [46]. By conditioning on the label *Y*, the bottleneck focuses compression on within-class nuisance factors (such as lighting, background, or canvas texture) without weakening the essential inter-style distinctions needed for accurate classification. Recent theoretical results demonstrate that controlling I(X;Z∣Y) yields strictly tighter generalization error bounds in supervised learning than penalizing unconditional mutual information [33]. Finally, this targeted regularization sharpens the optimization signal, each sample contributes directly to eliminating only its own within-class redundancy, resulting in more stable training and more interpretable latent representations.

There are two terms in Equation (4). Minimizing the negative of I(Y;Z) is equivalent to maximizing I(Y;Z). Note that I(Y;Z)=H(Y)−H(Y|Z), in which H(Y|Z) is the conditional entropy of *Y* given *Z*. Therefore,

(5) maximizeI(Z;Y)⇔minimizeH(Y|Z).

This is just because H(Y) is a constant that is irrelevant to network parameters.

Let p(x,y) denote the distribution of the training data, from which the training set ${\left\{x_{i},y_{i}\right\}}_{i=1}^{N}$ is sampled. Furthermore, let $p_{\theta }\left(z|x\right)$ and $p_{\theta }\left(y|z\right)$ denote the unknown distributions that we wish to estimate, parameterized by θ. We have [36]:

(6) $$H\left(Y|Z\right)≃E_{x,y∼p(x,y)}\left[E_{z∼p_{\theta }\left(z|x\right)}\left[-\log p_{\theta }\left(y|z\right)\right]\right].$$

We can, therefore, empirically approximate it by:

(7) $$\frac{1}{N}\sum_{i=1}^{N}E_{z∼p(z|x_{i})}\left[-\log p(y_{i}|z)\right],$$

which is exactly the average cross-entropy loss [47].

In this sense, our objective in Equation (4) can be interpreted as a classic cross-entropy loss (The same trick has also been used in the nonlinear information bottleneck [28], squared-nonlinear information bottleneck [28] and basic variational information bottleneck [9]) regularized by a weighted conditional mutual information term I(X;Z|Y). Hence, we name our framework the conditional information bottleneck (CIB).

### 3.2. Matrix-Based Entropy Functional and Its Gradient

The most challenging aspect of our CIB framework lies in accurately estimating I(X;Z∣Y). According to Shannon’s chain rule [48], $I\left(X;Z|Y\right)$ can be decomposed as:

(8) $$\begin{array}{cc} I\left(X;Z|Y\right)= & H\left(X|Y\right)-H\left(X|Z,Y\right)\\ = & H\left(X,Y\right)+H\left(Z,Y\right)-H\left(Y\right)-H\left(X,Z,Y\right), \end{array}$$

in which *H* denotes entropy or joint entropy.

In this work, instead of relying on variational approximation or using the popular mutual information neural estimator (MINE) [49], which may make the joint training becomes unstable or even result in negative mutual information values [50], we use the matrix-based Rényi’s α-order entropy functional [10,51] to estimate different entropy terms in Equation (8). This newly proposed estimator can be simply computed (without density estimation or any auxiliary neural network) and is also differentiable which suits well for deep learning applications. For brevity, we directly give the definitions.

**Definition 1.**  *Let κ:χ×χ↦R be a real valued positive definite kernel that is also infinitely divisible [52]. Given ${\left\{x_{i}\right\}}_{i=1}^{n}\in \chi$, each $x_{i}$ can be a real-valued scalar or vector, and the Gram matrix $K\in R^{n\times n}$ computed as $K_{ij}=\kappa \left(x_{i},x_{j}\right)$, a matrix-based analog to Rényi’s α-entropy can be given by the following functional:*

(9) $$\begin{array}{cc} H_{\alpha }\left(A\right) & =\frac{1}{1-\alpha }\log_{2}\left(\mathrm{tr}(A^{\alpha })\right)\\ & =\frac{1}{1-\alpha }\log_{2}\left(\sum_{i=1}^{n}\lambda_{i}{\left(A\right)}^{\alpha }\right), \end{array}$$

*where α∈(0,1)∪(1,∞). A is the normalized K, i.e., A=K/tr(K). $\lambda_{i}\left(A\right)$ denotes the i-th eigenvalue of A.*

**Definition 2.**  *Given a set of n samples ${\left\{x_{i},y_{i},z_{i}\right\}}_{i=1}^{n}$, each sample contains three measurements x∈χ, y∈γ and z∈ϵ obtained from the same realization. Given positive definite kernels $\kappa_{1}:\chi \times \chi \mapsto R$, $\kappa_{2}:\gamma \times \gamma \mapsto R$, and $\kappa_{3}:\epsilon \times \epsilon \mapsto R$, a matrix-based analog to Rényi’s α-order joint-entropy can be defined as:*

(10) $$H_{\alpha }\left(A,B,C\right)=H_{\alpha }\left(\frac{A\circ B\circ C}{\mathrm{tr}(A\circ B\circ C)}\right),$$

*where $A_{ij}=\kappa_{1}\left(x_{i},x_{j}\right)$, $B_{ij}=\kappa_{2}\left(y_{i},y_{j}\right)$, $C_{ij}=\kappa_{3}\left(z_{i},z_{j}\right)$, and A∘B∘C denotes the Hadamard product between the matrices A, B and C.*

Now, given ${\left\{x_{i},y_{i},z_{i}\right\}}_{i=1}^{M}$ in a mini-batch of *M* samples, we first need to evaluate three Gram matrices $K_{x}=\kappa \left(x_{i},x_{j}\right)\in R^{M\times M}$, $K_{y}=\kappa \left(y_{i},y_{j}\right)\in R^{M\times M}$, and $K_{z}=\kappa \left(z_{i},z_{j}\right)\in R^{M\times M}$ associated with variables *X*, *Y*, and *Z*, respectively. Based on Definitions 1 and 2, the entropy and joint entropy terms in Equation (8), all can be simply computed over the eigenspectrum of $K_{x}$, $K_{y}$, $K_{z}$, or their Hadamard product. Hence, our final estimator is expressed as:

(11) $$\hat{I}\left(X;Z|Y\right)=H_{\alpha }\left(K_{x},K_{y}\right)+H_{\alpha }\left(K_{z},K_{y}\right)-H_{\alpha }\left(K_{y}\right)-H_{\alpha }\left(K_{x},K_{z},K_{y}\right).$$

Throughout this work, we choose α=1.01 [10,51] and use the radial basis function (RBF) kernel $\kappa \left(x_{i},x_{j}\right)=\exp\left(-\frac{∥x_{i}-x_{j}{∥}^{2}}{2\sigma^{2}}\right)$ to obtain the Gram matrices. For each sample, we evaluate its *k* nearest distances and take the mean. We choose kernel width σ as the average of mean values for all samples. Interested readers are referred to Appendix A for additional details and a minimal PyTorch, version 2.1.0. implementation.

As can be seen, this new family of estimators avoids explicit estimation of the underlying data distributions, making it particularly attractive for challenging problems involving high-dimensional data. In practice, computing the gradient of $I_{\alpha }\left(A;C|B\right)$ is straightforward using any automatic differentiation framework, such as PyTorch [53] or TensorFlow [54]. We recommend PyTorch, as its computed gradients are consistent with the analytical ones.

**Figure 1:**
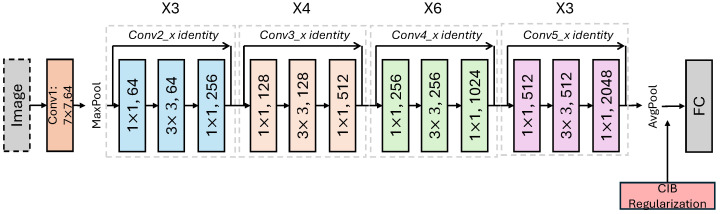
ResNet-50 with Conditional Information Bottleneck (CIB) Regularization. (X3), (X4), (X6) on top of the colored blocks refers to the number of times that particular residual block is repeated.

In our work, the bottleneck is inserted at a layer that still retains local spatial information. Specifically, for ResNet, it is added after the last block of the Conv5_x stage (see Figure 1).

## 4. Experimental Results

### 4.1. Dataset and Experimental Setup

**Figure 2:**
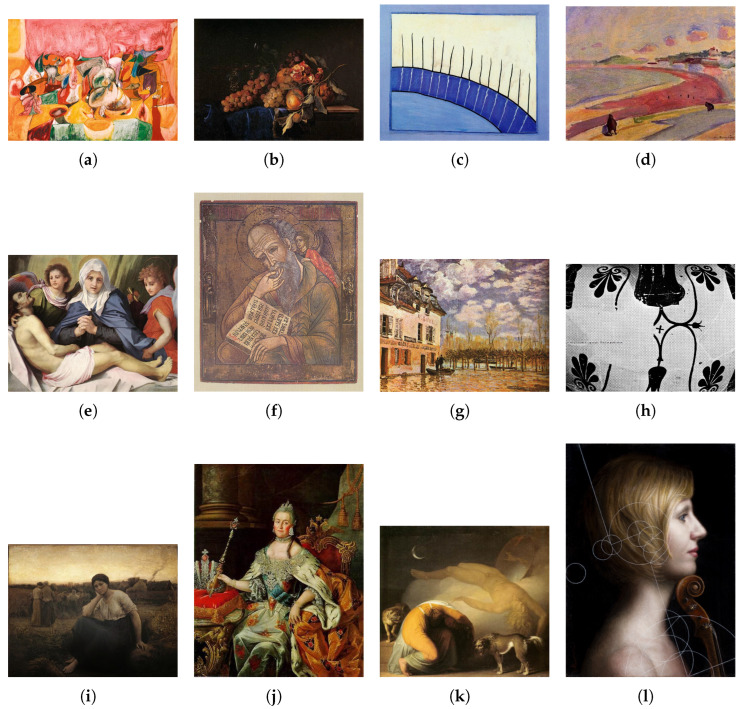
Exemplar images from Pandora dataset. (**a**) Abstract-Expressionism; (**b**) Baroque; (**c**) Cubism; (**d**) Fauvism; (**e**) High Renaissance; (**f**) Iconoclasm; (**g**) Impressionism; (**h**) OldGreekPottery; (**i**) Realism; (**j**) Rococo; (**k**) Romanticism; (**l**) Surrealism.

**Figure 3:**
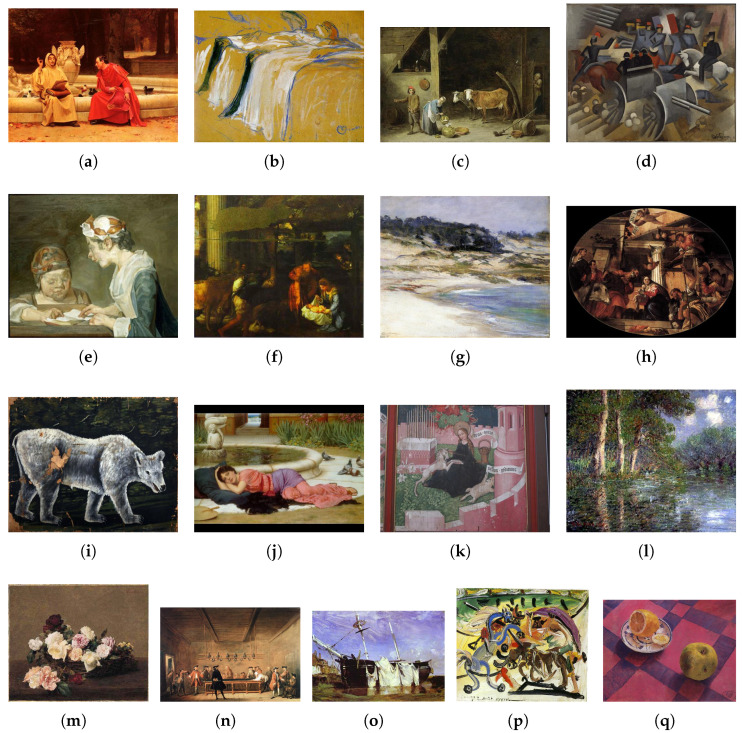
Exemplar images from OilPainting dataset. (**a**) Academicism; (**b**) Art Nouveau; (**c**) Baroque; (**d**) Cubism; (**e**) Expressionism; (**f**) High Renaissance; (**g**) Impressionism; (**h**) Mannerism; (**i**) Naive Art; (**j**) Neoclassicism; (**k**) Northern Renaissance; (**l**) Post-impressionism; (**m**) Realism; (**n**) Rococo; (**o**) Romanticism; (**p**) Surrealism; (**q**) Symbolism.

The oil-painting style classification experiments are conducted on two public benchmarks. The Pandora dataset contains 7740 images distributed across 12 distinct artistic movements (ranging from ancient Greek pottery to Romanticism) (http://imag.pub.ro/pandora/pandora_download.html (accessed on 1 March 2025)) [11] (see Figure 2), while the OilPainting dataset comprises 19,787 oil-painting images spanning 17 style categories (https://mmcv.csie.ncku.edu.tw/~wtchu/projects/OilPainting/index.html (accessed on 1 March 2025)) [12] (see Figure 3). Together, these datasets cover the full sweep of oil-painting stylistic evolution, capturing rich variation in color, brushstroke patterns, texture, and compositional layout.

For each dataset, we perform a random split of 80% of the images for training and 20% for testing. All images are resized to 224×224 pixels and normalized using ImageNet mean and standard deviation.

Our models are trained on an NVIDIA Tesla V100 GPU. We use a ResNet-50 backbone pre-trained on ImageNet and insert the information bottleneck module immediately after the last block of the third convolutional stage. In total, we compare six methods for style classification, including original ResNet-50, three information-theoretic approaches, and two additional architectural strategies designed to enhance generalization. The first is ResNet-50, which uses a standard classification head applied to the final global-pooling features. The second is variational information bottleneck (VIB) [9], which minimizes the mutual information I(X;Z) via a variational approximation using a Gaussian encoder. The third approach, deterministic information bottleneck (DIB) [13], achieves the same goal by employing a deterministic compression layer. Our proposed method, conditional information bottleneck (CIB), minimizes the conditional mutual information I(X;Z∣Y), using a matrix-based Rényi’s entropy estimator to capture class-specific discriminative features more effectively. We also compare with two additional approaches. The first is MSCNN [23], a multi-scale CNN that extracts features at different resolutions by applying the same CNN architecture on cropped and resized versions of the input image at multiple scales. The second is Ensemble CNN [25], which produces final predictions by aggregating the outputs of three networks, VGG-16, ResNet-50, and ResNet-19, using a meta-classifier to combine their predictions.

All models are trained for 100 epochs with a batch size of 32, using the Adam optimizer (initial learning rate $1\times 10^{-4}$, weight decay $1\times 10^{-5}$). We apply a cosine-annealing learning-rate schedule without restarts. For all IB methods, the trade-off parameter β (weight on the mutual-information term) is selected from $10^{-4}$ to 1. We evaluate performance using top-1 classification accuracy on the held-out test set and report the mean and standard deviation over three independent runs.

### 4.2. Generalization Performance

For generalization performance, we report the classification accuracy on the test sets of both datasets, as summarized in Table 1. We set the hyperparameter $\beta =1\times 10^{-2}$ for the ResNet-50 backbone in our method. The ADAM optimizer is used with an initial learning rate of $1\times 10^{-3}$, which is reduced by half every ten epochs. For other IB-based comparison methods, we adopt the β values as recommended in their original papers. As shown in Table 1, our method consistently outperforms existing IB approaches in terms of generalization performance across different test datasets.

### 4.3. Adversarial Robustness

Recently, various adversarial attack methods have been proposed to “fool” models by adding small, carefully designed perturbations. In this paper, we adopt two types of attack methods to evaluate adversarial robustness. The first is the standard baseline attack, the fast gradient sign method (FGSM) [55], which generates adversarial examples according to:

(12) $$\hat{x}=x+\epsilon \cdot \mathrm{sgn}\left(\nabla_{x}J\left(f_{\theta }\left(x\right),y\right)\right),$$

where *x* denotes the original clean image, ϵ is the perturbation magnitude, $\nabla_{x}J\left(f_{\theta }\left(x\right),y\right)$ is the gradient of the loss function with respect to the input *x*, and $\hat{x}$ represents the adversarially perturbed image.

The second attack method is projected gradient descent (PGD) [56], which generates adversarial examples through an iterative multi-step version of FGSM. The perturbed input at the *t*-th iteration, $x^{t}$, is updated as:

(13) $$x^{t}=\Pi_{\mathrm{clip}}\left(x^{t-1}+\alpha \cdot \mathrm{sgn}\left(\nabla_{x}J\left(f_{\theta }\left(x^{t-1}\right),y\right)\right)\right),$$

where $\Pi_{\mathrm{clip}}$ denotes the clipping function that constrains $x^{t}$ within a predefined perturbation range [−γ,γ], and α≤γ is the step size. The adversarial example $x^{t}$ is initialized as *x* and updated for *t* steps according to Equation (13). In our experiments, we set γ=0.1, α=0.02, and t=5 for PGD attacks. Note that, 5-step PGD attacks with a step size of 0.01 or 0.02 are commonly used in practice [38,56].

We evaluate adversarial robustness on both datasets under FGSM and PGD attacks, with results summarized in Table 2, Table 3, Table 4 and Table 5. As shown, our method consistently outperforms other IB approaches, particularly under the stronger PGD attack. This suggests that by retaining more task-relevant information while effectively compressing style-irrelevant details, our model achieves better resilience against adversarial perturbations.

### 4.4. Latent Space Visualization

**Figure 4:**
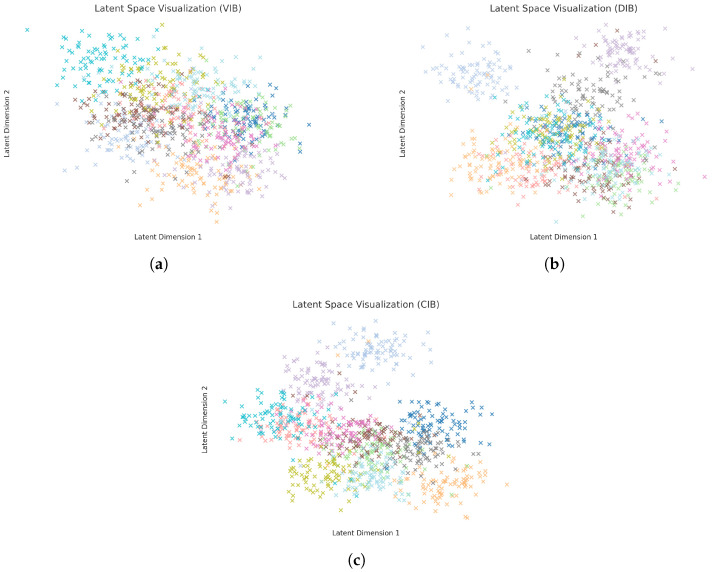
Latent space visualization of different methods on Pandora. (**a**) VIB; (**b**) DIB; (**c**) CIB. Different colors represent different categories.

**Figure 5:**
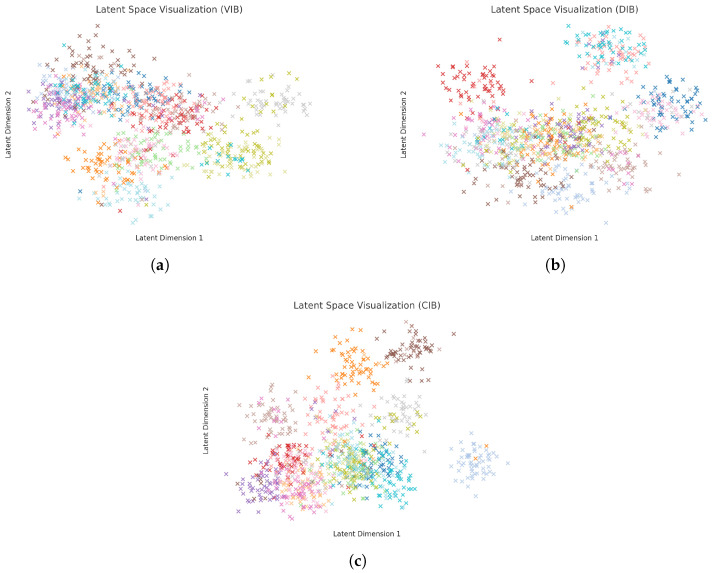
Latent space visualization of different methods on OilPainting. (**a**) VIB; (**b**) DIB; (**c**) CIB. Different colors represent different categories.

Finally, to better understand the structure of the learned representations, we visualize the latent spaces of the VIB, DIB, and our proposed CIB methods on both the Pandora and OilPainting datasets. Specifically, we extract the latent representations from the bottleneck layer and apply t-distributed stochastic neighbor embedding (t-SNE) [57] to project them into a two-dimensional space for visualization. Different colors are used to represent different style classes. The resulting visualizations are shown in Figure 4 and Figure 5.

As can be observed, for both datasets, the latent spaces produced by the standard VIB method exhibit substantial overlap between different style categories, indicating that VIB struggles to disentangle style-specific features effectively. The DIB method demonstrates moderate improvements, with partial class separation emerging, although notable overlaps still persist. In contrast, our CIB method yields a much cleaner and more structured latent organization, where samples from different style classes form compact and well-separated clusters.

These results suggest that minimizing the conditional mutual information I(X;Z∣Y) encourages the latent space to capture more task-relevant features while discarding style-irrelevant variations. The improved structure not only aligns with the observed gains in classification accuracy and adversarial robustness but also highlights the enhanced interpretability of the learned representations achieved by our CIB framework.

## 5. Conclusions

In this paper, we proposed a conditional information bottleneck (CIB) framework for oil-painting style classification, where the compression regularization explicitly minimizes the conditional mutual information (CMI) I(X;T∣Y). To estimate the CMI term, we adopt a matrix-based entropy functional, which avoids explicit density estimation and variational approximations, enabling stable and efficient training. Extensive experiments on two public datasets demonstrate that our CIB model consistently outperforms the standard variational information bottleneck (VIB) and deterministic information bottleneck (DIB) approaches, achieving higher classification accuracy and improved generalization performance. Furthermore, CIB enhances adversarial robustness, as the learned representations discard nuisance factors unrelated to style labels, making the classifier less sensitive to input perturbations. Qualitative analyses of the latent space show that our method produces more compact and class-aligned feature clusters, leading to greater interpretability compared to baseline bottleneck methods. These results validate the effectiveness of CIB in promoting both the utility and the interpretability of deep style representations in oil-painting analysis.   

## Figures and Tables

**Table 1:** Test accuracy (%) on Pandora and OilPainting datasets.

Method	Pandora	OilPainting
ResNet	57.30 ± 0.60	48.90 ± 0.55
MSCNN	60.10 ± 0.35	50.80 ± 0.50
Ensemble CNN	60.80 ± 0.30	51.20 ± 0.45
VIB	61.20 ± 0.35	51.80 ± 0.42
DIB	61.60 ± 0.28	52.10 ± 0.38
CIB (Ours)	64.80 ± 0.30	54.70 ± 0.40

**Table 2:** Test accuracy (%) under FGSM attacks with different perturbation strengths ϵ on the Pandora dataset.

Method	ϵ = 0	ϵ = 0.05	ϵ = 0.10	ϵ = 0.15	ϵ = 0.20
ResNet	57.3	50.2	44.1	38.0	32.5
VIB	61.2	54.7	48.5	43.2	38.0
DIB	61.6	55.5	49.8	44.7	39.5
CIB (Ours)	64.8	59.2	53.6	48.5	43.0

**Table 3:** Test accuracy (%) under PGD attacks with different perturbation strengths ϵ on the Pandora dataset.

Method	ϵ = 0	ϵ = 0.05	ϵ = 0.10	ϵ = 0.15	ϵ = 0.20
ResNet	57.3	47.1	38.9	32.0	26.3
VIB	61.2	51.3	42.0	34.8	29.5
DIB	61.6	52.5	43.6	36.4	30.1
CIB (Ours)	64.8	55.8	46.7	39.1	33.4

**Table 4:** Test accuracy (%) under FGSM attacks with different perturbation strengths ϵ on the OilPainting dataset.

Method	ϵ = 0	ϵ = 0.05	ϵ = 0.10	ϵ = 0.15	ϵ = 0.20
ResNet	48.9	42.0	36.1	30.4	25.1
VIB	51.8	45.2	39.6	34.2	29.1
DIB	52.1	45.7	40.2	34.8	29.8
CIB (Ours)	54.7	48.5	42.7	37.2	31.9

**Table 5:** Test accuracy (%) under PGD attacks with different perturbation strengths ϵ on the OilPainting dataset.

Method	ϵ = 0	ϵ = 0.05	ϵ = 0.10	ϵ = 0.15	ϵ = 0.20
ResNet	48.9	39.5	31.6	25.5	20.4
VIB	51.8	42.0	34.1	27.8	22.2
DIB	52.1	42.8	34.9	28.5	22.9
CIB (Ours)	54.7	45.2	36.8	30.0	24.3

## Data Availability

Data are contained within the article.

## Appendix A. Minimum Implementation of Conditional Mutual Information Regularized ResNet

Finally, we provide PyTorch implementation of our conditional mutual information estimator that avoids variational approximation, together with a training code of ResNet on Pandora data with 12 classes. Specifically, we show the code of the matrix-based Rényi’s α-order entropy functional as well as how to adaptively choose kernel width σ.

**Listing A1.** Conditional MI PyTorch.

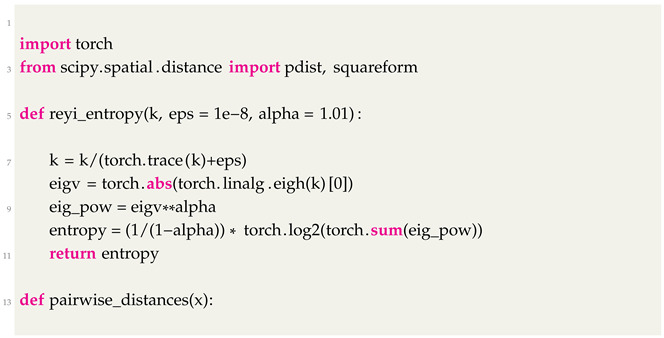

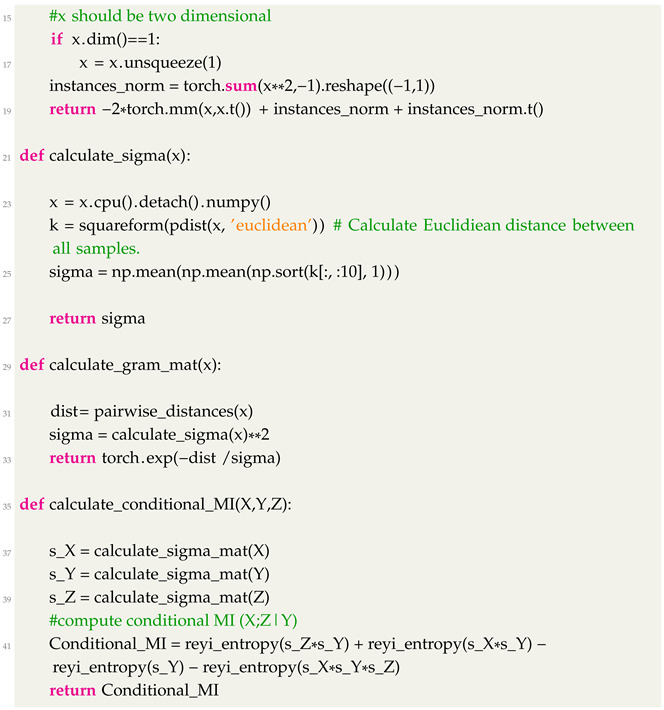

**Listing A2.** Training Conditional IB.

## References

[1] Karayev S., Hertzmann A., Trentacoste M., Han H., Winnemoeller H., Agarwala A., Darrell T. Recognizing Image Style. Proceedings of the British Machine Vision Conference 2014.

[2] Li W. (2025). Enhanced automated art curation using supervised modified CNN for art style classification. Sci. Rep..

[3] Mahmood T., Nawaz T., Irtaza A., Ashraf R., Shah M., Mahmood M.T. (2016). Copy-move forgery detection technique for forensic analysis in digital images. Math. Probl. Eng..

[4] Boccuzzo S., Meyer D.D., Schaerf L. (2024). Art Forgery Detection using Kolmogorov Arnold and Convolutional Neural Networks. arXiv.

[5] Bengio Y., Courville A., Vincent P. (2013). Representation learning: A review and new perspectives. IEEE Trans. Pattern Anal. Mach. Intell..

[6] Moyano M.Á.M., García-Aguilar I., López-Rubio E., Luque-Baena R.M. (2024). Improving Art Style Classification Through Data Augmentation Using Diffusion Models. Electronics.

[7] He K., Zhang X., Ren S., Sun J. Deep residual learning for image recognition. Proceedings of the IEEE Conference on Computer Vision and Pattern Recognition.

[8] Tishby N., Pereira F.C., Bialek W. The information bottleneck method. Proceedings of the 37th Annual Allerton Conference on Communication, Control and Computing.

[9] Alemi A.A., Fischer I., Dillon J.V., Murphy K. Deep variational information bottleneck. Proceedings of the International Conference on Learning Representations.

[10] Yu S., Sanchez Giraldo L.G., Jenssen R., Principe J.C. (2019). Multivariate Extension of Matrix-based Renyi’s *α*-order Entropy Functional. IEEE Trans. Pattern Anal. Mach. Intell..

[11] Florea C., Condorovici R., Vertan C., Butnaru R., Florea L., Vrânceanu R. (2016). Pandora: Description of a painting database for art movement recognition with baselines and perspectives. Proceedings of the 2016 24th European Signal Processing Conference (EUSIPCO).

[12] Chu W.T., Wu Y.L. Deep correlation features for image style classification. Proceedings of the 24th ACM International Conference on Multimedia.

[13] Yu X., Yu S., Principe J.C. (2021). Deep Deterministic Information Bottleneck with Matrix-based Entropy Functional. Proceedings of the IEEE International Conference on Acoustics, Speech and Signal Processing (ICASSP).

[14] Gao Z., Shan M., Cheong L.F., Li Q. (2015). Adaptive sparse coding for painting style analysis. Proceedings of the Computer Vision–ACCV 2014: 12th Asian Conference on Computer Vision.

[15] Liu X., Pu Y., Huang Y., Xu D. (2013). Quantitative statistics and analysis for painting visual art style. J. Front. Comput. Sci. Technol..

[16] Berezhnoy I., Postma E., van den Herik J. Digital analysis of Van Gogh’s complementary colours. Proceedings of the 16th Belgian-Dutch Conference on Artificial Intelligence (BNAIC’04).

[17] Qian W., Xu D., Xu J., He L., Han Z. (2019). Research on the classification of style painting based on information entropy. J. Graph..

[18] Bai S., Li P. (2023). Algorithm and simulation study of oil painting classification based on visual perception and improved embedded learning. J. Intell. Fuzzy Syst..

[19] Khan F.S., Beigpour S., Van de Weijer J., Felsberg M. (2014). Painting-91: A large scale database for computational painting categorization. Mach. Vis. Appl..

[20] Krizhevsky A., Sutskever I., Hinton G.E. (2012). Imagenet classification with deep convolutional neural networks. Adv. Neural Inf. Process. Syst..

[21] Folego G., Gomes O., Rocha A. (2016). From impressionism to expressionism: Automatically identifying van Gogh’s paintings. Proceedings of the 2016 IEEE International Conference on Image Processing (ICIP).

[22] Nanni L., Ghidoni S., Brahnam S. (2017). Handcrafted vs. non-handcrafted features for computer vision classification. Pattern Recognit..

[23] Peng K.C., Chen T. (2015). A framework of extracting multi-scale features using multiple convolutional neural networks. Proceedings of the 2015 IEEE International Conference on Multimedia and Expo (ICME).

[24] Kim J., Jun J.Y., Hong M., Shim H., Ahn J. (2019). Classification of oil painting using machine learning with visualized depth information. Int. Arch. Photogramm. Remote Sens. Spat. Inf. Sci..

[25] Menis-Mastromichalakis O., Sofou N., Stamou G. (2020). Deep ensemble art style recognition. Proceedings of the 2020 International Joint Conference on Neural Networks (IJCNN).

[26] Zhang X. (2024). Oil painting image style recognition based on ResNet-NTS network. J. Radiat. Res. Appl. Sci..

[27] Wang H., Liu X. (2022). Oil painting style classification based on deep learning. Proceedings of the 2022 IEEE 4th International Conference on Power, Intelligent Computing and Systems (ICPICS).

[28] Kolchinsky A., Tracey B.D., Wolpert D.H. (2019). Nonlinear information bottleneck. Entropy.

[29] Li H., Zhu C., Zhang Y., Sun Y., Shui Z., Kuang W., Zheng S., Yang L. Task-specific fine-tuning via variational information bottleneck for weakly-supervised pathology whole slide image classification. Proceedings of the IEEE/CVF Conference on Computer Vision and Pattern Recognition.

[30] Zheng K., Yu S., Li B., Jenssen R., Chen B. (2024). Brainib: Interpretable brain network-based psychiatric diagnosis with graph information bottleneck. IEEE Trans. Neural Netw. Learn. Syst..

[31] Mahabadi R.K., Belinkov Y., Henderson J. Variational Information Bottleneck for Effective Low-Resource Fine-Tuning. Proceedings of the International Conference on Learning Representations.

[32] Igl M., Ciosek K., Li Y., Tschiatschek S., Zhang C., Devlin S., Hofmann K. (2019). Generalization in reinforcement learning with selective noise injection and information bottleneck. Adv. Neural Inf. Process. Syst..

[33] Kawaguchi K., Deng Z., Ji X., Huang J. How does information bottleneck help deep learning?. Proceedings of the International Conference on Machine Learning.

[34] Kolchinsky A., Tracey B.D., Kuyk S.V. Caveats for information bottleneck in deterministic scenarios. Proceedings of the International Conference on Learning Representations.

[35] Poole B., Ozair S., Van Den Oord A., Alemi A., Tucker G. On variational bounds of mutual information. Proceedings of the International Conference on Machine Learning.

[36] Achille A., Soatto S. (2018). Information dropout: Learning optimal representations through noisy computation. IEEE Trans. Pattern Anal. Mach. Intell..

[37] Elad A., Haviv D., Blau Y., Michaeli T. Direct validation of the information bottleneck principle for deep nets. Proceedings of the IEEE International Conference on Computer Vision Workshops.

[38] Yu S., Yu X., Løkse S., Jenssen R., Principe J.C. Cauchy-Schwarz Divergence Information Bottleneck for Regression. Proceedings of the Twelfth International Conference on Learning Representations.

[39] Principe J.C. (2010). Information Theoretic Learning: Renyi’s Entropy and Kernel Perspectives.

[40] Shwartz-Ziv R., Tishby N. (2017). Opening the black box of deep neural networks via information. arXiv.

[41] Yu S., Giraldo L.G.S., Príncipe J.C. Information-Theoretic Methods in Deep Neural Networks: Recent Advances and Emerging Opportunities. Proceedings of the IJCAI.

[42] Chelombiev I., Houghton C., O’Donnell C. Adaptive Estimators Show Information Compression in Deep Neural Networks. Proceedings of the International Conference on Learning Representations.

[43] Yu S., Principe J.C. (2019). Understanding autoencoders with information theoretic concepts. Neural Netw..

[44] Yu S., Wickstrøm K., Jenssen R., Principe J.C. (2020). Understanding convolutional neural networks with information theory: An initial exploration. IEEE Trans. Neural Netw. Learn. Syst..

[45] Dong Y., Gong T., Chen H., Yu S., Li C. Rethinking Information-theoretic Generalization: Loss Entropy Induced PAC Bounds. Proceedings of the Twelfth International Conference on Learning Representations.

[46] Fischer I. (2020). The conditional entropy bottleneck. Entropy.

[47] Amjad R.A., Geiger B.C. (2019). Learning representations for neural network-based classification using the information bottleneck principle. IEEE Trans. Pattern Anal. Mach. Intell..

[48] MacKay D.J. (2003). Information Theory, Inference and Learning Algorithms.

[49] Belghazi M.I., Baratin A., Rajeshwar S., Ozair S., Bengio Y., Courville A., Hjelm D. Mutual information neural estimation. Proceedings of the International Conference on Machine Learning.

[50] Yu J., Cao J., He R. Improving subgraph recognition with variational graph information bottleneck. Proceedings of the IEEE/CVF Conference on Computer Vision and Pattern Recognition.

[51] Sanchez Giraldo L.G., Rao M., Principe J.C. (2014). Measures of entropy from data using infinitely divisible kernels. IEEE Trans. Inf. Theory.

[52] Bhatia R. (2006). Infinitely divisible matrices. Am. Math. Mon..

[53] Paszke A., Gross S., Massa F., Lerer A., Bradbury J., Chanan G., Killeen T., Lin Z., Gimelshein N., Antiga L. Pytorch: An imperative style, high-performance deep learning library. Proceedings of the Advances in Neural Information Processing Systems.

[54] Abadi M., Barham P., Chen J., Chen Z., Davis A., Dean J., Devin M., Ghemawat S., Irving G., Isard M. Tensorflow: A system for large-scale machine learning. Proceedings of the 12th USENIX Symposium on Operating Systems Design and Implementation (OSDI 16).

[55] Goodfellow I.J., Shlens J., Szegedy C. Explaining and harnessing adversarial examples. Proceedings of the International Conference on Learning Representations.

[56] Madry A., Makelov A., Schmidt L., Tsipras D., Vladu A. (2017). Towards deep learning models resistant to adversarial attacks. arXiv.

[57] Van der Maaten L., Hinton G. (2008). Visualizing data using t-SNE. J. Mach. Learn. Res..

